# Adolescents With Autism Spectrum Disorder and Anorexia Nervosa Comorbidity: Common Features and Treatment Possibilities With Cognitive Remediation Therapy and Oxytocin

**DOI:** 10.3389/fpsyt.2021.686030

**Published:** 2021-08-03

**Authors:** Barbara Plemeniti Tololeski, Ana Suhodolčan Grabner, Hojka Gregoric Kumperscak

**Affiliations:** ^1^Centre for Mental Health, Unit for Adolescent Psychiatry, University Psychiatric Hospital Ljubljana, Ljubljana, Slovenia; ^2^General Hospital Slovenj Gradec, Slovenj Gradec, Slovenia; ^3^Department for Child and Adolescent Psychiatry, University Medical Centre, Maribor, Slovenia; ^4^Faculty for Medicine, University of Maribor, Maribor, Slovenia

**Keywords:** autism spectrum disorder, anorexia nervosa, comorbidity, adolescents, cognitive remediation therapy, oxytocin

## Abstract

Autistic traits or autism spectrum disorder (ASD) can be found in 4% to 52% of anorexic patients, which makes the treatment of these patients very challenging. In this review, possible ways to treat ASD and anorexia nervosa (AN) comorbidity in children and adolescents are summarized. Over recent years, the focus has shifted from searching for the evidence of connections between these two disorders, which have started with Gillberg's study in 1983, to searching for more effective and holistic treatment of this comorbidity. The latter is known to contribute to more severe courses and worse prognosis, which is probably related to the obstacles in both diagnosing and treating. Since AN usually starts in early adolescence and high-functioning ASD children seem to begin struggling with increased pressure in adolescence, while various comorbidities can occur, it is important to improve the treatment of this comorbidity in young patients and to tailor it specifically in terms of diagnosing. In this paper, a literature review is conducted on common features and promising treatment possibilities. We describe cognitive remediation therapy and the promising pharmacotherapeutic candidate oxytocin with a special focus on adolescents.

## Introduction

Anorexia nervosa (AN) is a serious eating disorder, which is more often found in women and usually starts in adolescence. A study of a 30-year outcome of adolescent-onset AN has recently found that one-fifth of patients has had a chronic eating disorder (1). Many of these girls and young women seem to have problems in social functioning and have neurocognitive-specific features, which resemble the difficulties seen in autism spectrum disorder (ASD) patients. In 1983, Gillberg has been the first to find a high prevalence of autism in adult anorexic patients (2). Since then, many researchers have confirmed his results, finding autistic traits or ASD in 4–52% of anorexic patients according to the most recent review (3). What was once described as high-functioning autism (HFA) or Asperger syndrome (AS) seems to be harder to diagnose in women. The term HFA is an unofficial term used for people who would likely have been diagnosed with AS in the past. AS was removed from the Diagnostic and Statistical Manual of Mental Disorders in 2013 (4). Since then, the severity of ASD is indicated by a level from 1 to 3. According to DSM-5, people with HFA or AS would be diagnosed as ASD with a severity level 1, meaning that they can speak, read, write, and handle basic life skills and live independently. However, they still have difficulties in social interactions, conversations, including inflexibility of behavior and interest, and difficulty switching between activities, and are facing problems of organization and planning. In this paper, we continue to use the term ASD to cover the terms autism, HFA, and AS.

There are diagnostic difficulties in diagnosing AN in women with ASD. Due to the known camouflaging (5), diagnostic overshadowing (6), and partly also because of biases in assessment (7), many girls still pass by undiagnosed or misdiagnosed in childhood. Camouflaging is a process through which individuals mask autistic traits and is more common in girls and women than men (5). Diagnostic overshadowing explains a delay in diagnosing ASD in women with ASD symptoms being “overshadowed” by symptoms of comorbid disorder, such as eating disorder, depression, or anxiety (6). Often, it is only the comorbid disorder that refers a girl with ASD to the psychologist or psychiatrist for the first time. AN can be one of those, especially in early adolescence, when adolescents with ASD face normal developmental tasks, such as the adjustment to rapid and profound physical changes, development of stable and productive peer relationships, learning to manage their sexuality, and the need to develop expanded verbal skills to accommodate more complex concepts, which are even more challenging for them in comparison to neurotypical adolescents. Clinical practices have shown poorer outcomes for patients with this comorbidity (8), more severe clinical presentation on admission to inpatient care (9), and thus a need to introduce modified treatment of this comorbidity in young patients.

Recent data analysis on cognitive hallmarks in AN and ASD proposes common features, besides sensory sensitivities (10) and poor social functioning (11, 12), that contribute to the similarities in thinking in regard of these two disorders. There are indications that problems in cognitive flexibility and central coherence constitute the core for these cognitive features (13–16).

One target of research concerning the cognitive aspect in individuals with ASD and AN over the last years is cognitive remediation therapy (CRT) (17, 18). The assumption is that improving the cognitive function of the individual might enhance the ability to manage daily life tasks. The goal is to develop metacognition, i.e., teaching individuals to think about the way they think and focusing more on the process instead of the results (19, 20).

The other interesting target of recent research concerns the poor social functioning of young patients with this comorbidity. In the field of psychopharmacologic treatment, where there is still an absence of a specific drug for treating either AN or ASD, the use of oxytocin can be seen as a promising agent for augmentation, since it is known for its prosocial effects, from which these patients could benefit a lot.

Since treating this comorbidity poses quite a challenge and there are currently no clear guidelines, the South London and Maudsley NHS Trust Eating Disorders Services developed a new pathway, which was introduced in January 2018 to better assess the problems of ASD patients with an eating disorder. The Pathway for Eating disorders and Autism developed from Clinical Experience (PEACE pathway) provides a more customized approach toward treating the comorbidity of ASD and eating disorders (21), considering that this population of patients present with a more prominent clinical presentation and tend to have more trouble adhering to traditional treatment programs. As a result, outcomes are poorer and hospital admissions are longer (8, 9).

They may experience some additional issues when compared to neurotypical peers, such as communication difficulties, which may lead to more trouble engaging to treatment, sensory problems that subsequently motivate food restriction, and higher levels of cognitive rigidity, which makes it hard to modify maladaptive behaviors and cognitions (22, 23).

Several adaptations and improvements to the traditional approaches were made implementing the PEACE pathway, such as additional education of healthcare professionals in their multi-disciplinary teams leading to better tailoring of the treatment to the individual and more flexibility in their approach. Using questionnaires, like The AQ10 (Autism Quotient-10) (24), applying the updated algorithm to score the ADOS-2 (25), as it has been found to have increased sensitivity for females with AN (26), and using a sensory sensitivity scale that was given to all patients at the start of their treatment lead to better identification of possible autistic features in admitted patients (21). The patients were engaged in psychoeducation and experiential exercise-based cognitive and emotional training programs, using CRT and Cognitive Remediation and Social Skills Training (CREST) in individual and group formats immediately after admission, regardless of BMI. Therapeutic exercises were revised and adapted into a more concrete and tangible form (21). Observations showed that the patients presented with significant change in self-report and neuropsychological outcome after individual CRT and CREST. After group sessions, there were less significant changes in self report (27–29), although patients with high ASD traits experienced the groups as a safe, non-threatening environment, which might provide a good base for future tolerance of social situations (21).

In regard of addressing sensory sensitivities, the ward was tailored to the sensory needs of the patients and a specialized menu was co-produced by dieticians and patients. Clinicians received psychoeducation and support, which was also provided to the carers of their patients (21). A direct consequence of these adaptations was a clear reduction in the average duration of admission, average costs of admissions were lower, patients and carers gave positive feedback, while the confidence of the clinical team regarding treating patients with comorbid ASD and AN has increased (30).

There is evidence that CRT increases set-shifting abilities in individuals with AN and ASD features (27) and it presents a promising therapeutic approach. Nevertheless, there is still a gap regarding standardized treatment guidelines. Similarly, the poor functioning is one of the core symptoms in this comorbidity that seems to be important for etiopathogenesis, severity of disease, and prognosis. Oxytocin, which seemed to be of low levels in both anorexia and ASD according to some studies, is understood as an important promising candidate for augmentation treatment due to its involvement in social behavior and rewarding system in the central nervous system.

Treatment of ASD and AN comorbidity is very challenging and the data including children and adolescents are sparse. In this review article, we gathered information from most recent reviews and articles on the cognitive remediation techniques and oxytocin applied in individuals with AN and ASD traits and summarized findings from studies predominantly conducted over the last 6 years.

With reference to available data, both approaches, whether pharmacological or psychotherapeutic, seem to present a promising treatment option for this specific population of patients. Particularly regarding adolescents, the literature on this subject seems deficient; there are a limited number of studies that depict the implementation of oxytocin treatment or cognitive remediation techniques being used, but precisely the accessible literature presents a field worth of further exploration.

## Cognitive Remediation Therapy

CRT was first implemented for patients suffering from problems caused by brain lesions (31); however, over the years, it has been increasingly used as an intervention for various psychiatric illnesses (32–38). It targets cognitive domains such as attention, memory, visual and spatial functions, abstract and holistic thinking, and executive functions such as planning, cognitive flexibility, and problem solving in a way that it allows patients to embrace their weaknesses and strengths and reframe their thinking styles (27, 39).

Central coherence refers to the cognitive capacity to integrate information into a more global meaning with the assumption that executive functions and social cognition abilities are preserved (40). Weak central coherence presents itself as the preoccupation with details at the cost of global/contextual processing (17). Set-shifting abilities comprise the ability to flexibly switch through numerous mental tasks, change mindset, and, consequently, adapt more rapidly and respond to new situations or events (41). These abilities are of utter importance for cognitive and subsequently behavioral flexibility in everyday life. Weaknesses in set-shifting result in cognitive rigidity, and since it has been established that there are similarities in cognitive and behavior profiles in patients suffering from ASD or AN (10, 13, 16, 27, 42–45), the implementation of CRT techniques in patients with comorbid ASD and AN poses a valuable possibility of amelioration of these aberrations.

As mentioned before, there is a high comorbidity rate between ASD and AN, and over the last years, there have been several studies that support the usefulness of CRT techniques in AN and even some that see benefits for patients with ASD (17, 46–48).

### Cognitive Remediation in ASD

To date, there have been some randomized control trials regarding CRT interventions in ASD patients (49–52) as well as some relevant non-randomized control studies (53, 54), case series (27, 55–57), feasibility studies (58), and case studies (59) of which some also included children and adolescents (49–51, 55, 57).

It has been found that this particular form of therapy can be beneficial in improving cognitive flexibility, social cognition, and the heterogeneity of executive function, even clinical symptoms and working memory in autistic children as one study, including children and adolescent (age 6–21), established (55). On the other side, due to some methodological limitations of the studies conducted, it poses a question of generalizability of the submitted results (18).

Since individuals with ASD have trouble generalizing learned skills into real-life situations (56, 60), it might be worth considering modifying the standardized CRT interventions, such as setting more concrete examples, dismissing open ended questions, and implementing modeling, which may be helpful for learning, maximizing effectiveness, and reducing dropout rates. Perhaps a combination of CRT with social skills training might appear useful in solving difficulties in daily life (48).

On another important note, the trend of computer-based interventions is on the rise (53, 54); however, the research evidence regarding this is lacking and the interpretation of such results should be used with caution, particularly because it is difficult to translate these learned strategies into real-life situations (18).

### Cognitive Remediation in AN

Regarding the application of CRT interventions in populations of patients suffering from AN, there have been some naturalistic studies (46) conducted in the last years, as well as randomized control trials (47, 61–64), case series (65–78), and single case reports (79–81) mostly including adult populations alongside adolescents (47, 61, 63, 64, 77) or purely based on adults (46, 62, 73, 78–81), but also some being carried out on adolescents (67, 68, 70, 72, 75).

Through different cognitive exercises, metacognitive strategies, awareness, and their implementation in real-life situations, the aim of CRT in individuals suffering from AN is to improve the rigid and inadequate processing styles (67, 82). Despite several methodological challenges, the results appear to favor individual CRT, in that it was associated with lower dropout rates than group CRT and has the potential of creating a positive patient/therapist alliance. On the other hand, some positive aspect was also given to group CRT and family CRT but one should remain cautious because of the possibility of negative reinforcement in group dynamics as well as the fact that family CRT should not remain the only applied form of therapy. Instead, it should rather be used as an adjunct to other therapeutic procedures (17).

Some of the more recent research has validated similar cognitive processing styles in adolescents compared to adults (45, 83, 84), which would in turn pose a feasible ground for application of CRT techniques in the adolescent population.

Over the last years, applying CRT in younger adolescent patients with AN has shown improvements in central coherence and somewhat less consistently in set-shifting (66, 75, 85–87). There were even significant improvements in executive functioning observed (67, 75, 85, 86). Often, patients and therapists gave positive feedback (66, 69, 86, 88, 89) and low dropout rates were described (69, 86). Considering group CRT in adolescents with severe and complex eating disorders, there are some data about higher dropout rates; however, patients were generally satisfied with this kind of treatment approach (73, 86).

Nevertheless, it is worth acknowledging that improvements in set-shifting and central coherence following group CRT were more consistently documented in adult populations than in adolescents (14, 90, 91) as one recent systematic review established (10). The root of this issue seems to be the lack of research regarding adolescents, compared to the number of research done in adult populations (10). Despite the fact that most RCTs to this point were conducted on adult populations with AN, there is an indication for positive outcome following individual (66, 75), group (73), and family-based CRT in adolescents suffering from AN (70, 71).

Another reasonable subject to consider is using CRT as an adjunct or pre-treatment option for more extensive psychotherapeutic work in cases of acute AN (79, 80, 92) and as an enhancement of the current therapeutic measures for patients with an enduring eating disorder (61).

There are studies that show that using CRT in addition to medical stabilization for adolescent inpatients with AN could aid greater patient motivation to change and thus reduce hospital admission duration. It also enhances the cognitive skills needed for more extensive psychotherapy, and there have been positive changes noted in neurocognitive functioning following CRT in combination with other psychotherapeutic approaches (39, 86). Studies to further investigate the feasibility of CRT interventions in this domain are currently taking place (20, 93).

### Cognitive Remediation in ASD and AN

There has been little research on the clinical effects of CRT in patients with comorbid ASD and AN and basically no relevant data on CRT implementation in adolescents who suffer from both ASD and AN (27, 48, 56, 59). Even in the present studies, some of the participants with AN did not have a valid ASD diagnosis and were only assessed using screening tools and self-report questionnaires (18).

By taking a closer look into the results of these studies, there were some contradictory findings in the central coherence improvement as two studies reported positive changes (48, 59), one study found no statistically significant improvements in central coherence (18), and in one of the studies, there were no significant changes in self-reported thinking styles after group CRT or improvements in motivation in the group of patients with high ASD traits, although patients gave positive feedback on the applied techniques (56).

After a trial of individual CRT, patients self-reported positive changes in cognitive flexibility and an improvement in mean scores for cognitive flexibility was found; however, there were no significant changes found in using the performance-based measure for cognitive rigidity (the Brixton Test) (18).

In the only case study discussing the clinical implementation of CRT for AN in ASD in a 21-year-old female patient (59), there were also some improvements noted in cognitive flexibility, alongside with an increase in body mass index (BMI).

Regarding the form of CRT delivered for this comorbidity, group treatment showed less improvements following therapy, which may be due to the possibility that patients with high ASD features need some adjustment to the standard type of cognitive training delivery and all in all a modified individual approach of CRT would eventually be more suitable (56).

Considering the neurocognitive similarities in individuals with ASD and AN, discussed in this article, CRT certainly seems like a promising technique to incorporate into existing treatment approaches of these patients. The prosperity of group CRT for comorbid adolescents should warrant additional investigation, precisely because of the unique thinking patterns, emotional processing, and behavioral features of this patient population. It would also be important to explore the long-term effects of CRT in adolescents with ASD and AN by applying measurements for facing everyday challenges or quality of life.

Data on implementing CRT in patients with comorbid ASD and AN, particularly younger populations, remains scarce, and although it gives rise to some positive outcomes, further research is needed (19, 48, 56, 59).

## Oxytocin

### Why Oxytocin?

Oxytocin is a natural hormone and neurotransmitter with a broad spectrum of functions in animals and humans, being known colloquially as the “love hormone.” Though many other pharmacotherapeutic agents have an important role in the treatment of both AN and ASD and their comorbidities, oxytocin has drawn the attention of researchers as promising medicine in psychiatry and somatic medicine years ago.

In regard to the absence of an approved medication for AN or ASD, it is one of the novel possible agents studied in these two diseases as well, especially due to the recognized impaired emotional–social processing in ASD and AN patients. One of its probably most important effects in mammals, apart from being involved in sexuality, labor, and lactation, is the induction of prosocial behavior. As such, it can induce bonding (94) and enhanced empathy (95). It also shows anxiolytic properties in humans (96) and antidepressant-like effects in animals (97), which can be beneficial in ASD, since depressive and anxious disorder is commonly comorbid. It is also important in stress, showing protective effects (98) with lowering concentrations of cortisol (99). The study from 2018 (100) showed that the density of oxytocin receptors in ventral pallidum, an area in the mesolimbic reward pathway, decreases with age. They also found the peak of oxytocin receptor density in that area in early childhood in neurotypical children, which suggests that early childhood is a critical period for social development in humans.

Oxytocin also has a special role in feeding with its anorexigenic effects, which were also proven in experimental setting in animals, where a single dose of oxytocin reduced the feeding, but did not have an effect on food intake in humans (101).

In the last decade, there has been an increasing amount of literature devoted to testing its positive effects through intranasal administration in patients with different psychopathology. Below, we review studies on the use in AN and ASD with special emphasis on studies examining adolescents, which are unfortunately still scarce; therefore, it is difficult to draw definite conclusions.

### Oxytocin in AN

AN is one of the most difficult mental disorders to treat, especially in children and adolescents where, due to its egosyntonic nature in early age, there is no or poor insight. Regarding the absence of FDA-approved medication, our pharmacotherapeutic attempts to help patients with AN symptoms and its comorbidities include the use of SSRI, atypical antipsychotics, such as olanzapine and benzodiazepines, frequently in combination. In his recent review, Frank (102) suggests future studies of combinations of more medications used together, combinations of medications and psychotherapy, and some novel agents, especially those targeting dopamine system in conjunction with medications to modulate GABA neurotransmission.

The beforementioned anorexigenic effects of oxytocin seem to exclude it from the list of possible agents for treating AN. However, according to recent findings, oxytocin is probably involved in etiopathogenesis of AN. Tyszkiewicz-Nwafor et al. (103) conducted a study on adolescents with AN and found elevated oxytocin concentrations that insisted even after partly weight restitution. Their findings are contradictory to a series of studies that found lower oxytocin concentrations in patients with AN (104). A correlation between peripheral levels and central activity is still questionable (104).

Interestingly, lower oxytocin levels are supposed to be associated with increased severity of social–emotional functioning impairment and greater severity of alexithymia in AN (105), which could be the consequence of lower insula activity due to lower oxytocin levels (106). The pathology of insula has been proven to be connected to alexithymia (107). According to a study from 2020, alexithymia is thought to be important in pathogenesis and treatment of eating disorders in autistic and neurotypical individuals (108).

Although studies from the previous decade showed promising effects of intranasal oxytocin in anorexic patients, e.g., with attenuated attentional bias for fat stimuli and increased emotion recognition (109), recent systematic review and meta-analysis (110) warn of potential shortcomings of those studies. Oxytocin also had no effect on the amount of drink intake in two studies (111, 112). However, lowered cortisol concentrations after meal and oxytocin administration was found in both serum (112) and saliva (113), which potentially could help to ease the anxiety linked to meals in anorexic patients.

However, oxytocin receptor polymorphism seems to be important too. It was found to be related to the severity of eating disorders with alleles for two SNPs (rs53576 and rs22542988) identified as being responsible for a more severe course of anorexia (114). The study from 2018 (115) showed that OXTR SNP rs2254298 could be involved in modified neural response on social stimulus in AN.

As seen, the results of the studies done mainly on adult population are unambiguous, starting with differences in oxytocin levels in AN from low to increased. The concentrations of oxytocin in the central nervous system after intranasal administration may also not be sufficient (110). More studies on adolescents are required in the future due to the possible effect of disease duration and adaptation mechanisms in adult patients and to provide more answers to the state/trait question regarding altered oxytocin in AN.

### Oxytocin in ASD

Oxytocin is seen as a promising psychopharmacological candidate for augmenting ASD regarding its prosocial effects and the role in emotional–social processing. Moreover, it may be even more significant in childhood and adolescence due to an age dependence of oxytocinergic system (100). In the study (100) of post-mortem brain tissue of individuals with ASD and neurotypical individuals, they found that individuals with ASD lacked a peak of density of oxytocin receptors in ventral pallidum in early childhood, which might explain a deficit in social functioning in autism due to impaired connections between prosocial behavior and reward system. Early childhood seems to be a critical period with oxytocinergic system involved in the etiology of ASD. The study in mice showed that oxytocin administration in a critical development phase prevented social deficits later in life of mice with a mutated gene that was also found in patients with ASD (116). Nevertheless, the results of research are still quite contradictory, from low oxytocin levels found in children with ASD (117) to the varying ones (118). The complexity of oxytocinergic system can be imagined through findings such as increased levels of inactive form of oxytocin found in children with ASD (119), large heritability of oxytocin levels (114), oxytocin receptor gene polymorphism being related to the symptom severity in ASD (114), and the importance of oxytocin receptor gene methylation as epigenetic regulation. The latter was shown to be linked to a decrease in the extent to which social information captures attention, which could be important in ASD etiology (120). A recent study (121) found that extreme values of DNA methylation in the first intron of oxytocin receptor gene correlate with a lower IQ and more social problems in individuals with ASD and ADHD.

After intranasal administration of oxytocin in adolescents with autism, they found improved emotion recognition (122); however, when administered twice daily for 8 weeks, no improvements were observed in social behavior in adolescents with ASD (123). On the other side, some of the studies found improved social communication in children and adolescents with ASD after longer periods of oxytocin administration (124, 125). In recent years, they also measured changes in brain with fMRI after administration of oxytocin in studies, some of them conducted on the adolescent population (126–128), which could be more objective in comparison to the ones measuring oxytocin effects by behavioral findings [reviewed in (129)].

In summary, oxytocin seemed to enhance the processing of social information and facilitate reward responses in autistic individuals. In this recent review from 2020 (129), authors suggest that future research should try to maximize the effects of oxytocin by creating more personalized dosing schedules, considering initial basal levels of endogenous oxytocin in individuals as well as factors such as sex, comorbid disorders, and others. Furthermore, future research should investigate the combination of oxytocin administration and behavioral intervention, since social stimuli can be more rewarding for individuals with ASD after oxytocin administration resulting in a more efficient behavioral intervention (129).

### Oxytocin in AN and ASD Comorbidity?

Treating children and adolescents with AN and ASD comorbidity by applying intranasal oxytocin is still not possible according to the latest research on oxytocin administration in both conditions as seen above. However, this field should be further investigated in the future to keep oxytocin as a promising candidate for augmentation in treatment. Improvement of social impairments in this group of patients could mean a lesser extent of AN chronification. As suggested by Baker (129), future studies should investigate the efficiency of combination of oxytocin administration and behavioral interventions in ASD patients. In the light of a modern concept of precision psychiatry, the individuals with lowered oxytocin levels should be detected to benefit the most. More studies on oxytocin administration in this population burdened with AN and ASD should be conducted in the future.

Alexithymia could be a new focus in future research. It occurs in half of the autistic individuals and is greatly connected with their social functioning. It is understood as at least partly responsible for a greater risk for any eating disorder (108). With the knowledge that lower oxytocin levels are linked with decreased activity in insula (106), thus causing more alexithymia, we can hypothesize that further studies should keep oxytocin as a candidate in the treatment for this comorbidity. Childhood and adolescence seem to be the most sensitive time for the development and maturing of the oxytocin system, as well as for the social inputs from the environment. Therefore, combining oxytocin administration and behavioral interventions could result in less impairment in social functioning in adult age and life-quality improvement. Of course, further studies on youth are required in the future, including genetics, neuroimaging, and combined pharmacological and behavioral techniques, as proposed by Baker (129) for ASD.

## Discussion

Treating young patients with ASD–AN comorbidity has been improving in recent years due to better and earlier recognition of ASD, especially in girls as well as the progress in the development of interdisciplinary programs for youth with ASD. The examination of recent literature shows that among other interventions and therapies, young patients with AN and ASD could also benefit from CRT and oxytocin application.

An advantage of CRT is its flexible and modifiable approach, not only regarding the form of delivery, meaning application in an individual, family, or group scheme, but also intervention-wise to best fit the individual in treatment (20). Future implementation should aim toward a modification of CRT techniques, making them more adapt to the deviations in social functioning in patients with ASD.

The use of oxytocin, on the other hand, still needs to be proven in more studies in this group of patients. According to the studies already done, we can conclude that oxytocin should stay a possible psychopharmacologic candidate, which could help to prevent chronification of AN, due to its promising ability to improve their poor social functioning. Future studies should combine oxytocin administration together with behavioral interventions, as proposed by Baker (129) for ASD patients, which could be even more true for patients with AN and ASD comorbidity.

Applying CRT therapy and treating comorbid patients with oxytocin presents a favorable new ground for treatment and research, also because there were no reports on the important side effects of intranasal administration of oxytocin (130) or CRT. Still, there are some limitations to consider. Using stricter patient inclusion criteria and using standardized and up-to-date diagnostic questionnaires for ASD when selecting the research groups of potential patient candidates would contribute to more relevant end results.

This review shows that studies on treatment of ASD–AN comorbidity are still sparse, especially on adolescents. Studies on CRT and oxytocin in adolescents are very rare, so drawing ultimate conclusions of its usefulness might still be premature. Future studies should focus on young patients with this comorbidity, combining psychotherapy and pharmacotherapy. The approach of precision medicine should be undertaken due to known individual differences on autism spectrum, gender differences, and many comorbidities in ASD children and adolescents. It would be necessary to include larger treatment centers into conducting more studies with a larger patient sample. Particularly, more RCTs featuring adolescents are needed to assess the effectiveness of CRT and use of oxytocin in this age group. By doing so, this may possibly lead to earlier discovery of comorbid features, a more suitable and thorough therapeutic approach leading to less recurrent hospital admissions, a lower dropout rate, and fewer psychiatric comorbidities.

Until more studies are done, we suggest to follow the South London and Maudsley NHS Trust Eating Disorders Services first guidelines regarding the treatment of comorbid patients with ASD and AN. The PEACE pathway (21, 30) was published with comprehensive adaptations to treatment and set a promising foundation for future therapeutic work. In view of the positive outcomes, this approach could also be used as a base for the potential implementation in children and adolescents with comorbid ASD and AN. Further investigation is warranted, with future studies featuring the PEACE pathway guidelines, thus gathering more data of the benefits that it might provide.

## Author Contributions

BP and AS: study design, collection and assembly of data, data interpretation, and contributed to the manuscript equally. HK: study design, article review, revision, and final approval of the article. All authors contributed to the article and approved the submitted version.

## Conflict of Interest

The authors declare that the research was conducted in the absence of any commercial or financial relationships that could be construed as a potential conflict of interest.

## Publisher's Note

All claims expressed in this article are solely those of the authors and do not necessarily represent those of their affiliated organizations, or those of the publisher, the editors and the reviewers. Any product that may be evaluated in this article, or claim that may be made by its manufacturer, is not guaranteed or endorsed by the publisher.
